# Genetic and genomic analyses of testicular hypoplasia in Nellore cattle

**DOI:** 10.1371/journal.pone.0211159

**Published:** 2019-01-24

**Authors:** Haroldo H. R. Neves, Giovana Vargas, Luiz F. Brito, Flavio S. Schenkel, Lucia G. Albuquerque, Roberto Carvalheiro

**Affiliations:** 1 GenSys Associated Consultants, Porto Alegre, Rio Grande do Sul, Brazil; 2 Department of Animal Sciences, School of Agricultural and Veterinary Sciences, São Paulo State University (UNESP), Jaboticabal, São Paulo, Brazil; 3 Department of Animal Sciences, Purdue University, West Lafayette, Indiana, United States of America; 4 Centre for Genetic Improvement of Livestock (CGIL), Department of Animal Biosciences, University of Guelph, Guelph, Ontario, Canada; 5 National Council for Science and Technological Development (Cnpq), Brasília, Distrito Federal, Brazil; University of Florida, UNITED STATES

## Abstract

Reproductive performance is a key indicator of the long-term sustainability of any livestock production system. Testicular hypoplasia (TH) is a morphological and functional reproductive disorder that affects bulls around the world and consequently causes major economic losses due to reduced fertility rates. Despite the improvements in management practices to enhance performance of affected animals, the use of hypoplastic animals for reproduction might contribute to expand the prevalence of this disorder. The aim of this study was to identify genomic regions that are associated with TH in Nellore cattle by performing a genome-wide association study (GWAS) and functional analyses. Phenotypic and pedigree data from 47,563 animals and genotypes (500,689 Single Nucleotide Polymorphism, SNPs) from 265 sires were used in this study. TH was evaluated as a binary trait measured at 18 months of age. The estimated breeding values (EBVs) were calculated by fitting a single-trait threshold animal model using a Bayesian approach. The SNP effects were estimated using the Bayes C method and de-regressed EBVs for TH as the response variable (pseudo-phenotype). The top-15 ranking windows (5-adjacent SNPs) that explained the highest proportion of variance were identified for further functional and biological network analyses. The posterior mean (95% highest posterior density) of the heritability for TH was 0.16 (0.08; 0.23). The most important genomic windows were located on BTA1, BTA3, BTA4, BTA5, BTA9, BTA22, BTA23, and BTA25. These windows explained together 22.69% of the total additive genetic variance for TH. Strong candidate genes associated with metabolism and synthesis of steroids, cell survival, spermatogenesis process and sperm motility were identified, which might play an important role in the expression of TH. Our findings contribute to a better biological understanding of TH and future characterization of causal variants might enable improved genomic prediction of this trait in beef cattle.

## Introduction

Beef production is a large component of the Brazilian economy and over time various investments have been made by producers, breeding companies and research institutions to increase the industry production efficiency and profitability. For instance, the implementation of the Special Certificate of Identification and Production (CEIP) in 1995 by the Brazilian Ministry of Agriculture, Livestock Production and Food Supply to identify and trace genetically superior beef animals has contributed substantially to improve genetic progress in Brazilian beef cattle herds. The CEIP is issued to producers involved in the data recording and genetic evaluation systems, in which only 20 to 30% of the top animals, evaluated at weaning and yearling, are eligible to receive this certificate. After the traditional genetic evaluation, these animals are evaluated for morphological and functional defects or disorders to confirm their eligibility to receive the CEIP. These phenotypes include feet and leg problems, prognathism and agnathism, nasal deviation, depigmentation and testicular hypoplasia (TH). Despite of having a high genetic merit, animals with these defects or disorders will have reduced reproductive and productive performance as well as welfare issues. In addition, these undesirable phenotypes could be transmitted to their progeny, which could increase the problem and, consequently, the economic losses.

Testicular hypoplasia is among the main genetic and congenital disorders in cattle, and its incidence can influence the genetic progress and profitability of livestock industries. The main genetic causes of TH are considered to be aneuploidy, chromosomal abnormalities and the expression of a recessive autosomal gene with incomplete penetrance, interfering on spermatogenesis process [1, 2]. This disorder is characterized by incomplete development of the germinal epithelium of the seminiferous tubules, due to inadequate numbers of germinal cells within the testis, leading to a decrease in spermatic concentration and increased morphological alterations of spermatozoids, as well as azoospermia [3].

The affected animals can present uni- or bilateral, partial or total TH [4]. Partial unilateral TH is characterized by decreased of 1.5 to 2.0 cm in one of the testicles, usually the left side one. Partial bilateral TH is defined by a reduced size of both testicles, and consequently reduced scrotal circumference. Unilateral total TH is characterized by a large difference in size between testicles, and bilateral total TH is characterized by reduced size of both testicles accompanied by complete sterility. Animals with unilateral TH usually have normal reproductive rates, i.e., their libido and ability to breed are not affected, but the reproductive life is shorter compared to non-affected animals [5]. Diagnosis of TH can be done visually and based on physical examination of bulls at 7 to 9 months of age [1]. Hypoplastic animals present flaccid testicular consistency caused by poor development of sperm-producing tissue and reduced size of testicles. The diagnosis of this disorder can be done more accurately through ultrasonography by comparing echogenicity of both testicles.

Efforts to better understand the genetic architecture of TH have been done in different cattle populations [6, 7]. However, these results need to be validated in independent populations and to our knowledge there are no studies investigating TH in Nellore cattle. Thus, the main objectives of this study were: 1) to perform a genome-wide association study (GWAS) to detect genomic regions associated with TH in Nellore cattle; and, 2) to perform comprehensive functional analyses of TH to identify and better understand the candidate genes and metabolic pathways associated with the expression of this disorder.

## Material and methods

Animal care and approval of the ethics committee of the School of Agricultural and Veterinary Sciences, São Paulo State University (UNESP) were not necessary as all the data used here was obtained from pre-existing databases.

### Phenotypic and pedigree data

Phenotypic and pedigree information of 47,563 Nellore cattle from the Alliance Nellore database (www.gensys.com.br) collected between 2005 and 2013 were used for this study. The animals belonged to herds that systematically perform evaluations for morphological and functional defects or disorders. Testicular hypoplasia was evaluated as a binary trait measured at 18 months of age. Contemporary groups (CG) were defined by concatenating CG at weaning, farm at yearling, management group at yearling, and measurement date. The CG at weaning were defined by concatenating season of birth, farm at birth, management group at birth, and farm at weaning. Data from CG with fewer than 10 records and/or without variability in the trait were removed from further analyses. Connectedness among CG was determined by using the AMC software [8] and only CG with at least 10 genetic links were kept for the analyses. After editing, 46,256 phenotypic records were kept for the analyses.

### Genotypic data and quality control

A total of 8 Nellore animals were genotyped using the Illumina BovineSNP50 Genotyping BeadChip (50K; Illumina, Inc., San Diego, CA, USA) and 257 animals were genotyped using the Illumina BovineHD Genotyping BeadChip (HD; Illumina, Inc., San Diego, CA, USA). All animals had a genotype call rate greater than 90%. The FImpute v2.2 software [9] was used for genotype imputation from the 50k SNP chip to the HD SNP chip. The genotyping quality control (QC) filtered out markers that were located on non-autosomal regions, mapped at the same position, deviated from Hardy-Weinberg equilibrium (HWE) test (P<10^−5^), had SNP call rate lower than 0.98 and minor allele frequency (MAF) less than 0.01. Samples with call rates lower than 0.90 and duplicated samples were also removed from the dataset. The remaining number of SNPs after QC was 500,689. The QC analyses were performed using customized scripts combining *Unix* and *awk* commands and PLINK v.1.07 software [10].

### Genetic parameter estimation

The variance components and estimated breeding values (EBVs) for each animal was obtained through Bayesian analyses by fitting a single-trait threshold animal model using the Gibbs sampling program THRGIBBS1F90 [11]. The statistical model used in the analysis can be described in matrix notation as:

l=Xβ+Za+e (1)

where **l** is a vector of underlying liabilities of TH; **β** is a vector of fixed systematic effects (**CG** and age of animal at recording as covariate); **a** is a vector of random additive direct genetic effects (breeding values); **e** is a vector of random residual effects, and **X** and **Z** are incidence matrices that relate the liabilities in **l** to the effects in **β** and **a**, respectively. It was assumed that $a∼N(0,A\sigma_{a}^{2})$ and $e∼N(0,{I\sigma }_{e}^{2})$, where **A** is the numerator relationship matrix, $\sigma_{a}^{2}$ is the additive genetic variance, **I** is an identity matrix and $\sigma_{e}^{2}$ is the residual variance.

The underlying liabilities of TH were defined as:

$$\begin{array}{c} \{y_{i}=0\;if\;l_{i}\leq t_{1};\\ y_{i}=1\;if\;l_{i}>t_{1}\}; \end{array}$$

in which: y_i_ is the score for the i^th^ animal, t_1_ corresponds to threshold that define, on the underlying scale, the mutually exclusive categories of TH (0 or 1) [12]. In the analysis, the parameterization $\sigma_{e}^{2}$ = 1 was adopted. A single chain with a length of 300,000 cycles was generated, applying a conservative burn-in period of 30,000 cycles and a thinning interval of 50 cycles. The convergence of the chains was assessed using the Geweke’s [13] test, in addition to visual inspection.

Approximated genetic correlations were estimated between TH and 17 traits/indexes used as selection criterion in the studied population using the procedure proposed by Calo et al. [14]. According to this procedure, Pearson’s correlations are estimated between the EBVs of the animals and then adjusted based on the respective accuracies of the EBVs. Estimated breeding values for the 17 traits/indexes were obtained from routine analyses of Alliance Nelore database (www.gensys.com.br).

### Genome-wide association analyses

The EBVs were de-regressed using the method proposed by Garrick et al. [15] and used as dependent variables to estimate SNP effects for TH. The reliabilities of EBVs required for the deregression process were obtained based on posterior mean of additive variance and posterior variances of EBVs obtained from each chain. Under the assumptions of normality and known covariance structure of the model, posterior variances of the EBVs are the variances of prediction error [16]. Only the dEBVs with reliability greater than 0.10 were used in the analyses.

The Bayes C method [17] was used in order to estimate SNP effects for TH. The Bayes C method allows produce shrinkage and variable selection, including all markers simultaneously, assuming distinguish prior distributions, being widely suggested as a suitable method for QTL detection [18]. The Bayes C method consisted of fitting a mixture model for SNP effects and can be described as:

$$y=1_{n}\mu +Zg+e$$

where **y** is the vector of pseudo-phenotypes (dEBVs); **μ** is the overall mean; **1**_***n***_ is a the vector of 1’s, **Z** is the incidence matrix relating animals to pseudo-phenotypes; **g** is the vector of breeding values and **e** is the vector of random residuals. It was assumed that $e∼N(0,{R\sigma }_{e}^{2})$, where **R** is a diagonal matrix, whose elements account for the differences in the reliabilities of the observations in **y**, similarly as in VanRaden [19]. The diagonal elements of **R** (R_ii_) were obtained as $R_{ii}=(1-r_{i}^{2})/(r_{i}^{2})$, where $r_{i}^{2}$ is the reliability associated with the i^th^ dEBV.

The elements of vector **g** were calculated for each animal as: $g=\sum_{j=1}^{n}\left(z_{j}a_{j}I_{j}\right)$, in which **z**_**j**_ is the vector containing the coded genotypes of the animals for the j^th^ SNP; **a**_**j**_ is the allele substitution effect of the j^th^ SNP; and **I**_***j***_ is an indicator variable (equal to 1 if the j^th^ SNP has a non-zero effect on the trait and 0 otherwise).

Model parameters were estimated within a Bayesian framework. It was assumed that a_j_ ~ N(0, $\sigma_{a}^{2}$) and e_i_ ~ N (0, $R_{ii}\sigma_{e}^{2}$). Scaled inverse chi-squared distributions, with v degrees of freedom and scale parameter S were assumed for $\sigma_{a}^{2}$ and $\sigma_{e}^{2}$. Thus, it was assumed in this mixture model that SNP marker effects are sampled from a single (Normal) distribution. An arbitrarily small value was assumed for v (v = 4), while the scale parameters were derived according to Habier et al. [17]. A binomial distribution with probability (1-**π**) was assumed for I_j_ and an informative *beta* distribution was assigned for **π** (implying that this parameter was kept constant about 0.01).

The Bayes C was performed using the Markov Chain Monte Carlo (MCMC) algorithm implemented in the software GS3 [20], running a single chain with 100,000 iterations, a burn-in period of 20,000 and a thin interval of 50.

### QTL mapping and functional annotation analyses

The results of GWAS are reported as the proportion of variance explained by a window of 5 non-overlapping adjacent SNPs. These windows were further investigated by searching genes and QTLs reported in the QTLdb database [21] located within the same genomic regions, using the bovine genome assembly UMD3.1.1 [22]. The presence of genes and QTL in adjacent windows (100 kb to the left and to the right of the top-15 windows) were also investigated, as their effect can be captured by neighboring SNPs due to linkage disequilibrium. Annotated genes in the top-15 windows were identified using the National Center for Biotechnology Information (NCBI) Map Viewer tool (www.ncbi.nlm.nih.gov/mapview/). Manhattan plots were created using the R package “*qqman*” [23].

Annotation was performed using Blast2GO software [24] (http://www.blast2go.de/). All assembled putative genes were searched against the sequences in the NCBI non-redundant protein (NR) database using the BLASTp algorithm to retrieve GO (Gene Ontology) terms and annotation to select reliable functions. Three basic steps are needed to GO annotation in Blast2GO: homologues search, GO term mapping, and actual annotation. The default for statistical significance threshold (e-value threshold of 1e-03) for reporting matches against database sequences and a maximum of 20 blast hits were used. According to this criteria, statistical significance greater than the expect threshold do not have the match reported. Sequences with a BLAST match were mapped and annotated. InterProScan was used to verify the GO terms assigned via BLAST. The Kyoto Encyclopedia of Genes and Genomes (KEGG) [25, 26] (http://www.genome.jp/kegg/pathway.html) was used to identify metabolic pathways associated with significant sequences.

The STRING database [27] was used to annotate protein-protein associations among the candidate genes. For each association a score (i.e., the edge weight in each network) is provided, indicating the estimated likelihood that a specific interaction is biologically meaningful [28]. The interactions are represented by seven different ‘evidence channels’ which are assigned by edges of different color: (1) experiments channel: evidence from experiments in the lab; (2) database channel: evidence that has been stated by a human expert curator; (3) textmining channel: based on searching of protein names that have been mentioned in different sources, such as PubMed abstracts and text collections [29]; (4) coexpression channel: pair of proteins presenting similar expression patterns [30]; (5) neighborhood channel: genes receive an association score when they are presented in each other’s genome neighborhood; (6) fusion channel: genes receive an association score when there is one organism where their orthologs have fused into a single, protein-coding gene; (7) co-occurrence channel: the phylogenetic distribution of orthologs of the proteins are evaluated [31].

## Results

### Estimation of genetic parameters

Table 1 presents the summary statistics of the data used in the quantitative analysis of TH obtained after preliminary data editing. The incidence of animals presenting this disorder was 4.61%. The posterior means (95% highest posterior density) of direct additive genetic and residual variances, and heritability estimate obtained for TH were 0.19 (0.09; 0.31), 1.01 (0.99; 1.03) and 0.16 (0.08; 0.23), respectively.

**Table 1:** Summary statistics of testicular hypoplasia data set in Nellore cattle.

Factor	Summary
Number of observations	46,256
Number of sires	1,307
Number of contemporary groups	1,616
Number of males	46,167
Average age of the animals at measurement	536.14
Percentage of affected animals	4.61

**Fig 1:**
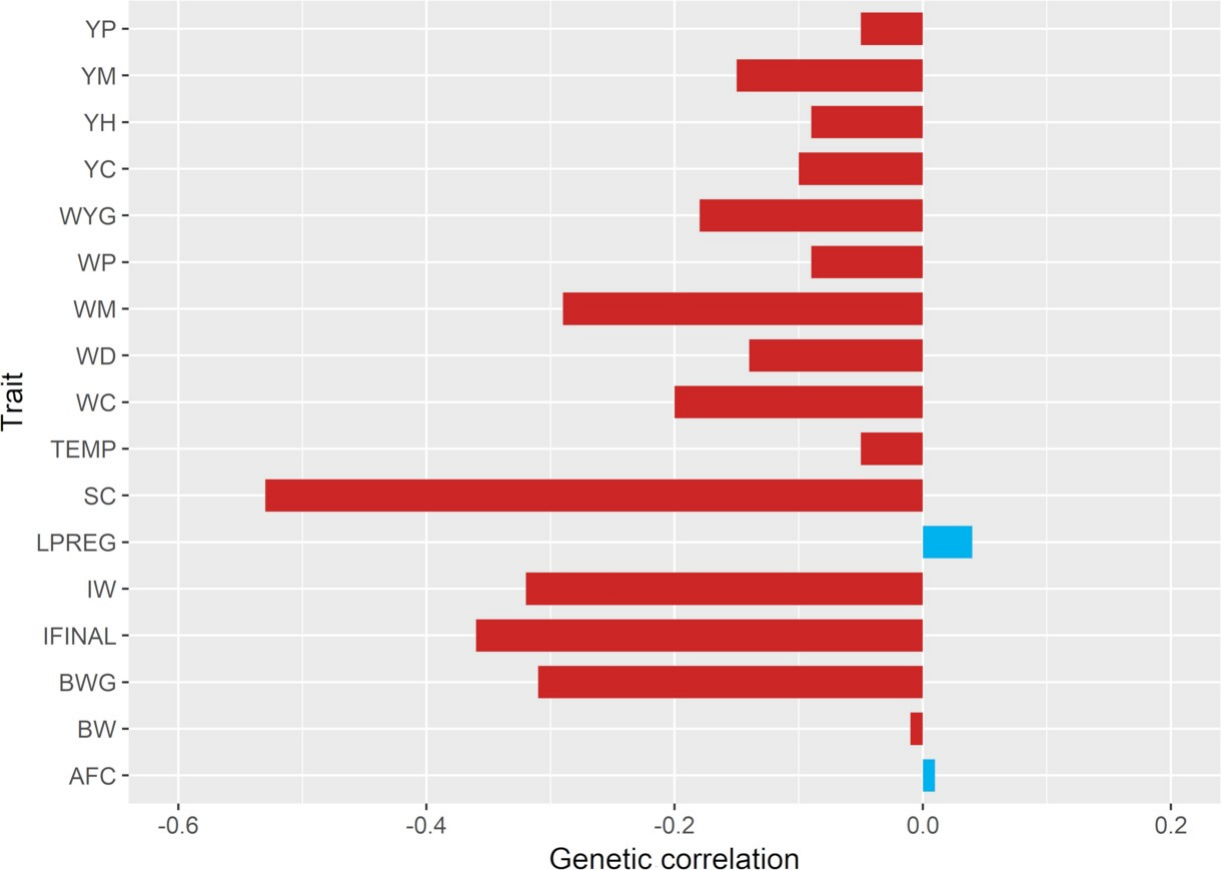
Estimates of genetic correlations between testicular hypoplasia and the traits/indexes used as selection criteria in the studied population. BWG: birth-to-weaning weight gain; WYG: weaning-to-yearling weight gain; WC, WP and WM: conformation, finishing precocity and muscling score at weaning, respectively; YC, YP and YM: conformation, finishing precocity and muscling score at yearling, respectively; BW: birth weight; YH: height at yearling; WD: adult weight of cow; TEMP: temperament; SC: scrotal circumference; LPREG: length of pregnancy; IW: selection index at weaning; IFINAL: selection index combining traits at weaning and yearling; AFC: age at first calving.

Fig 1 shows the genetic correlations for TH and the 17 traits/indexes used as selection criteria in the studied population. Genetic correlations ranged from -0.53 to 0.04. A moderately negative genetic correlation was obtained between scrotal circumference and TH (-0.53). Negative (favorable) correlations were also observed between TH and the selection index at weaning (-0.32) and the selection index combining weaning and yearling traits (-0.36).

### Pseudo-phenotypes (De-regressed EBVs)

A total of 265 Nellore bulls, with progeny evaluated for TH, were genotyped. The mean and standard deviation (SD) of dEBVs were equal to 0.01 and 1.34, respectively. The reliabilities of dEBVs ranged from 0.10 to 0.92, with an average reliability equal to 0.33.

### Genome-wide association study

**Fig 2:**
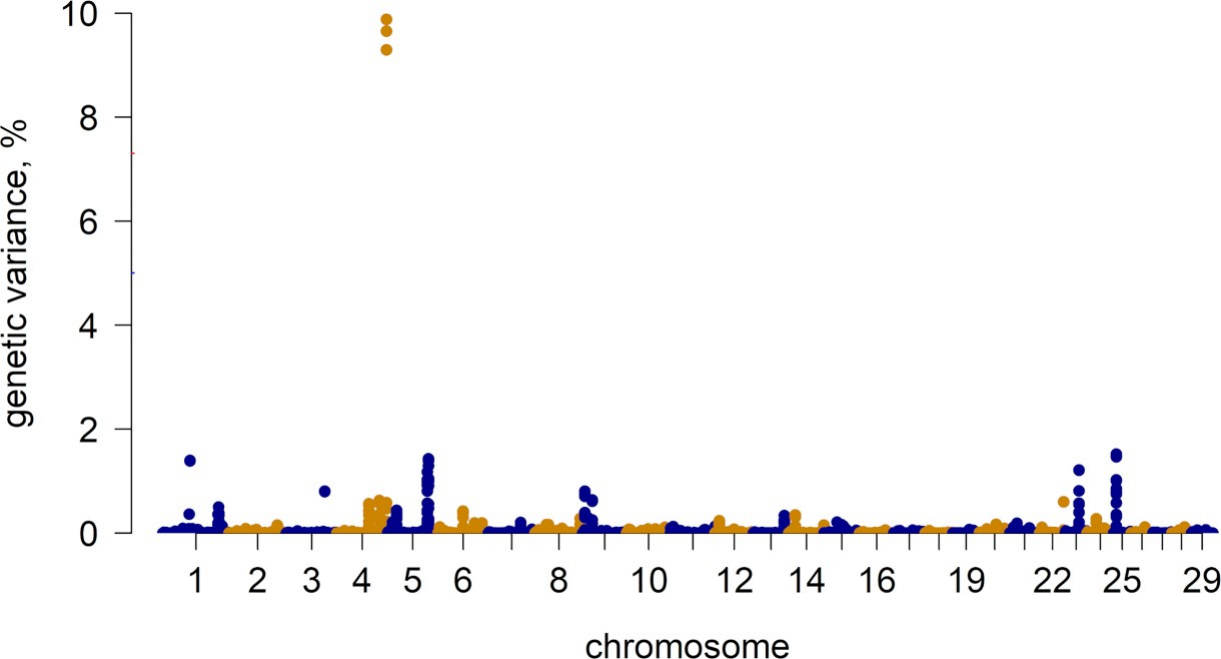
Manhattan plot for testicular hypoplasia in Nellore cattle.

Fig 2 shows the Manhattan plot with the percentage of additive genetic variance explained by 5 adjacent SNP windows across the 29 chromosomes for TH. The top-15 genomic regions with large effects obtained for TH are located on BTA1, BTA3, BTA4, BTA5, BTA9, BTA22, BTA23, and BTA25, and together explained 22.69% of the total additive genetic variance (Table 2). The most important window was located on BTA4 (between 117.95 and 117.96 Mb) and explained almost 10% of the total genetic variance.

**Table 2:** Annotated genes within the top-15 windows of 5 adjacent SNPs that explained the highest proportion of genetic variance for testicular hypoplasia in Nellore cattle.

Chr	Position (bp)	Genes	Pvar
4	117,949,070–117,960,312	*PAXIP1*, *INSIG1*, *EN2*, *CNPY1*	9.88
25	7,028,236–7,033,242	-	1.52
5	97,928,246–97,941,767	*BORCS5*, *MANSC1*, *LRP6*, *LOC104972539*	1.43
1	64,466,014–64,473,854	*IGSF11*, *C1H3orf30*	1.40
23	32,459,521–32,481,143	*MIR2285AD*, *LOC788595*, *LOC537017*, *RIPOR2*	1.21
5	95,126,670–95,131,355	*PTPRO*, *RERG*	1.17
25	7,054,169–7,065,411	-	0.87
9	5,410,553–5,429,252	-	0.81
3	92,024,587–92,043,440	*TMEM61*, *DHCR24*, *LEXM*, *TTC22*, *PARS2*, *TTC4*, *MROH7*	0.81
9	22,816,140–22,831,920	-	0.64
4	100,710,381–100,716,618	*MTPN*	0.64
22	57,040,083–57,047,493	*IFT122*, *MBD4*, *EFCAB12*, *RPL32*, *RF00409*, *CAND2*, *TMEM40*, *RAF1*	0.61
23	32,507,191–32,525,190	-	0.57
4	76,151,685–76,167,465	-	0.57
1	133165988–133178288	*RF00026*, *IL20RB*	0.50

Chr: chromosome; Position (bp): starting and ending coordinates; Pvar: % genetic variance explained by the SNPs within the window

Three out of the top-15 genomic regions associated with TH have already been reported within Quantitative Trait Loci (QTL) associated with reproductive traits. These regions have been reported to be associated with scrotal circumference and structural soundness traits in cattle, and were located on BTA1 (between 64.37 and 64.57 Mb), BTA9 (between 22.72 and 22.93 Mb), and BTA23 (between 32.46 and 32.48 Mb) [32, 33].

A total of 34 positional candidate genes were found for TH (Table 2). The window harbouring the largest number genes (eight genes) was located on BTA22. The main biological processes obtained from gene ontology (GO) enrichment analyses were involved in biological regulation, cellular, localization, metabolic, multicellular organismal, and response to stimulus processes. Pathway analysis enabled the identification of genes involved in the “purine metabolism”, “steroid biosynthesis”, aminoacyl-tRNA biosynthesis”, “biosynthesis of antibiotics”, and “thiamine metabolism” pathways.

**Fig 3:**
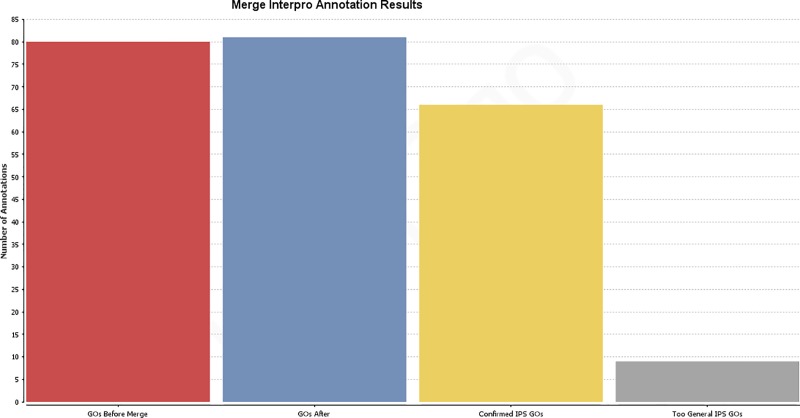
Results distribution after implementation of InterProScan results. GOs before merge: total number of added GO terms after Blast2GO annotation; GOs after: total number of GO annotations after implementation of InterProScan results; Confirmed IPS GOs: number of initial GO annotations confirmed by InterProScan result; Too general IPS GOs: number of GO annotations removed after InterProScan because of a lack of specificity.

A total of 81 GO terms were assigned after merging primary GO annotations with the InterProScan results (Fig 3).

**Fig 4:**
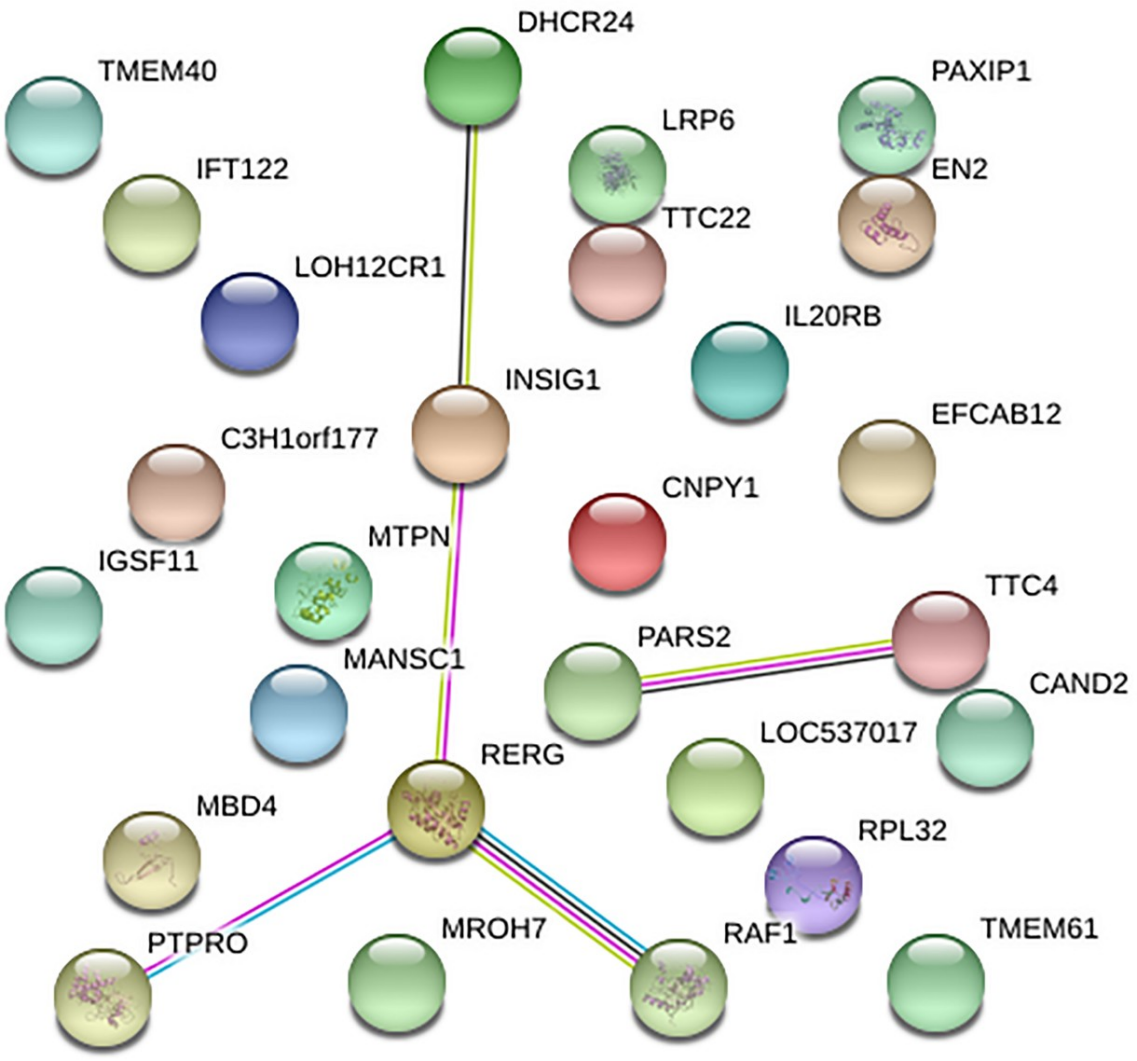
Protein network of candidate genes for testicular hypoplasia in Nellore cattle, according to STRING action view. Nodes represent proteins; edges and arrows indicate interaction.

The STRING data base allowed to identify known and predicted protein interactions from the candidate genes identified for TH (Fig 4). The functional partnerships and associations that occur between proteins are represented by edges, which are meant to be specific and meaningful, being therefore possible to characterize molecular biological systems in which the genes are involved. A total of 5 genes (out of 26) from three different windows were connected through gene networks for TH.

## Discussion

The incidence of animals presenting TH disorder was about 5%, indicating possible substantial economic losses, because the animals are unable to receive a CEIP and subsequently, be sold as sires. The heritability estimate obtained for TH was of considerable magnitude (0.16), suggesting that these traits are under genetic control and should respond to selection. In general, genetic correlation estimates between TH and the 17 traits/indexes used as selection criteria in the studied population ranged from low to moderate magnitude. As expected, considerable negative genetic correlation was obtained between TH and scrotal circumference (-0.53), since this latter is an indirect measurement of the sperm-producing capacity of the testicles and it is highly correlated with testis weight [34]. The negative correlation means that animals with lower scrotal circumference have higher probability of presenting TH. Animals with TH are characterized by presenting small scrotal circumference due to a lack of germinal epithelium, resulting in a reduced production of normal semen [3].

Genome-wide association study results showed in the Manhattan plot (Fig 2) indicated the presence of peaks on BTA1, BTA4, BTA5, BTA9, BTA23, and BTA25, suggesting a polygenic nature for TH. Some of the top-15 windows identified had been previously associated with scrotal circumference (BTA1:64.37 to 64.57 Mb, and BTA9:22.72 to 22.93 Mb) in cattle [33]. There were no overlapping genomic regions with a study by Venhoranta et al. [7], in which the authors performed a GWAS for gonadal hypoplasia in Northern Finn cattle and Swedish Mountain cattle. According to the authors, the translocation of ~500kb chromosomal segment from BTA6 to BTA29 is likely to be the underlying genetic mechanism responsible for gonadal hypoplasia.

The genomic window located on BTA4 that explained the highest percentage of additive genetic variance (9.88%) harbors genes involved in the metabolism and synthesis of steroids. The *INSIG1* is a gene that plays an important role in the feedback control of lipid synthesis via its sterol-dependent binding to sterol regulatory element-binding proteins (SREBPs) [35]. SREBPs are a family of membrane-bound transcription factors that enhance transcription of genes encoding cholesterol and fatty acid biosynthetic enzymes and the LDL receptor [36]. Male steroid hormones are mainly synthesized from cholesterol in the testicular *Leydig* cell and play important roles in spermatogenesis [37]. Another interesting gene located in this genomic region is the PAX Transcription Activation Domain Interacting Protein 1 Like gene (*PAXIP1)*. The *PAXIP1* complex has an important role on cell survival process in response to DNA damage. This finding could help to better understand the occurrence of TH, since one of the primary causes of this trait is chromosomal abnormality [1, 2]. In addition, *PAXIP1* acts as transcriptional cofactor for nuclear hormone receptors and inhibits the induction properties of several steroid receptors.

The BTA1 (64.46 to 64.47 Mb) and BTA5 (95.12 to 95.13 Mb) regions identified here also contain important candidate genes for TH. The immunoglobulin superfamily 11 (*IGSF11)* was previously described as a gene preferentially expressed in testis [38]. The *IGSF11* gene was identified in *Sertoli* cells in mice and its under-expression resulted in male infertility, atrophic testicles, azoospermia, and spermatogenesis arrest [39]. The Ras-like estrogen regulated growth inhibitor (*RERG*) belongs to the mammalian Ras superfamily members that are involved in the regulation of several cellular function processes. Previous studies have reported its expression in several tissues, such as adrenal, thyroid, and testis, in humans and mice [40, 41]. Interestingly, the adrenal gland is one of the numerous organs that are known to have the capacity to synthesized biologically active steroids [42], indicating a possible association of this gene with the TH occurrence.

The genomic window located on BTA22 between 57.04 and 57.05 Mb contains a gene that belongs to an intraflagellar transport protein family, the *IFT122*, associated with assembly and maintenance of cilia and flagella. This family has previously described as related with male fertility and spermiogenesis (spermatide elongation) in mice, being involved in the transport cargo proteins for sperm flagella formation [43]. Sperm flagella is a motile microtubule-based structure required for normal sperm motility and dysfunctions in this structure may lead to failure of fertilization.

Biological network analysis allowed the identification of interactions between the positional candidate genes for TH (Fig 4). Some of these genes, depicted as interacting with others, were previously discussed in relation to their functional association with TH. The *INSIG1* gene showed an interaction with *DHCR24* and *RERG* genes, which are known to be expressed in the testicles and are involved in the steroid synthesis [40, 41, 44]. The *TTC4* gene that was also part of the gene network has its ubiquitous expression in testicles and encodes a protein that contains tetratricopeptide repeats (TPR). TPR has an important role on physiological processes related to steroid hormone activity [45].

Regarding to the pathway analysis, an important result is the identified “Steroid biosynthesis” pathway. Sperm production is directly influenced by steroid biosynthesis including follicle-stimulating hormone (FSH) involved in spermatogenesis process and luteinizing hormone (LH), which are related to production of testosterone [46]. Furthermore, the sperm concentration is positively associated with testicular length, diameter and scrotal circumference [47]. Hypoplastic animals usually present complete absence of germinal epithelium, with only *Sertoli* cells in the seminiferous tubule interior. This compromises the spermatogenesis and mitosis of spermatogonia, since they are essential in the conversion of testosterone into estradiol [48].

Another pathway identified as associated to TH is the “Aminoacyl-tRNA biosynthesis” pathway, which involves the “serine and threonine metabolism”. Serine/threonine kinase are enzymes used to compose the glycogen synthase kinase-3 (GSK3), an enzyme regulated by phosphorylation related to motility initiation in the epididymis and regulation of mature sperm function in bovine [49]. Previous study have also reported that an isoform of this enzyme, the GSK3α, could play an unfavorable role in the regulation of porcine sperm motility and suggested that the sperm motile quality might to be regulated according the activity state of the GSK3α [50].

The identification of genomic regions associated with TH provides a better understanding of the genetic background of this trait in cattle. From the top-15 genomic regions identified as potentially associated with TH, the window on BTA4 explained most of the total genetic additive variance, which highlights the importance of this genomic region to the expression of TH.

## Conclusions

A genome-wide association study using a high-density SNP panel identified genomic regions and positional candidate genes associated with testicular hypoplasia in Nellore cattle. Furthermore, several functional and biological processes likely involved in the testicular hypoplasia disorder, such as metabolism and synthesis of steroids, cell survival and spermatogenesis process, supported the evidence that these genomic regions are associated with testicular hypoplasia in Nellore cattle. The metabolic pathways and regulatory mechanisms identified in this investigation may contributed to a better understanding of testicular hypoplasia disorder in future studies. Future characterization of causal variants might enable improved genomic prediction of this trait in Nellore cattle.
